# Internal Antiseptics

**Published:** 1895-06-08

**Authors:** Tom R. Taylor


### INTERNAL ANTISEPTICS.

By Tom R. Taylor, M.D., B.S., B.Sc. (Lond.) F.R.C.S.Eng.

So great, so magnificent, in fact, have been the results of antiseptics applied to external lesions, that great hopes have been raised of their successful internal administration, especially in diseases which are distinctly known to depend upon the action of micro-organisms. But here we have great and important obstacles to contend with. In the first place, nearly every one of these important drugs is poisonous, destroying not only the lower forms of life, but also acting in an injurious or even fatal manner upon the tissues of those of higher organisation. They can, therefore, only be administered in very small doses, and the extent of their dilution becomes extreme.

In the second place, it must be remembered that once administered internally a chemical substance soon passes under the sway of the forces of living protoplasm. And that while some actually enter into new combinations and become inert in stomach or intestines, others pass into the tissues, and there undergo changes and set up reactions vastly different to any reproducible in laboratory experiments. At the same time, in certain conditions much good has resulted from an application of the general principles of antisepsis, and from the zeal with which fresh inquiries are being instituted, greater success may be safely augured for the future. The most striking results of the direct application of antiseptics internally is seen in those conditions which arise directly from decomposition or fermentation. As in the huge dilated bladders, with their ammoniacal urine, found in cases of old stricture and enlarged prostate, where the injection of dilute antiseptic solutions not only controls and finally suspends these processes, but relieves the general symptoms which they evoke, so in certain stomach cases great good may arise from a similar treatment.

In this category we must place the treatment introduced by Professor Kussmaul for dilated stomach. No one who for any length of time has been connected with a hospital can fail to have seen the excellent results, and especially the comfort of the patient, brought about by the washing out of the stomach, especially when to the water originally used some mild antiseptic such as creasote in small quantity has been added.

In cases where the pylorus is obstructed the ingesta collect, and passing through the outlet very slowly fermentation takes place. By washing out the viscus the products of this process are removed, and by its systematic employment the yeast-like scum and sarcinæ, so often found, may be quickly reduced to a minimum.

This, the direct application of the great principle of cleanliness and the direct washing out of the stomach, naturally leads us to those agents which retard and actually prevent fermentation. Flatulent dyspepsia, where troublesome eructations take place either soon or some time after meals, is greatly benefited by the internal administration of phenol or creasote, which checks the fermentative processes taking place in the viscus. Dr. Fuller speaks highly of phenol in such cases.

M. Lemaire and other French authorities prescribe phenol with water 1-1,000. Lemaire recommends 7½ grains as a dose for adults. This is to be dissolved in two tumblers of sugared water and taken four hours after a meal and two hours before the next. These directions would certainly require some modification in England.

The direct action of internal antiseptics is seen in the remarkable effects of phenol and the sulphocarbolates upon the urine. Urine from animals to which sulphocarbolates had been administered has been preserved without undergoing decomposition for as long as six months (Sansom).

Excellent results have been obtained where it has been merely a question of ordinary decomposition and fermentation, and where it has been possible to bring antiseptics directly into contact with the saprophytic organisms. But in the case of pathogenic germs results have not been so encouraging.

The meagre knowledge we possess up to the present of the nature of the virus in many diseases believed to be due to bacillar influence, must be taken into account, and also the almost universal distribution of the germs in others with whose pathology we are better acquainted. Perhaps in no disease have more methods of treatment been tried than in England's scourge, phthisis. Here hopes have been raised of dealing directly with the tubercle bacilli in the lung.

Medicaments have been given internally in the hope of reaching the materies morbi by means of the blood stream.

Iodoform has been given. The great germicidal effect of iodine upon the tubercle bacillus, and the marked effect of the drug when directly applied to external tubercular lesions strongly recommended it. But the desired results have not been attained. Balsams have certainly been found to have a beneficial effect where expectoration is abundant and offensive, but the disease has continued its progress.

Much has been said of respirators for the inhalation of various aromatic substances, *e.g.*, eucalyptus oil, &c. Improvement has been recorded, and would that by this simple and pleasant means recovery could be hoped for! But it is at the best palliative.

Miquel and Rueff recommend direct spraying of lungs and bronchi with a solution of perchloride of mercury one gramme, iodide of potassium one gramme, and water 1,000 grammes. They record (Traitement de la Tuberculose Pulmonaire) twenty-seven cases which they have treated in this manner, and claim improvement in nineteen, estimated by diminution of physical signs, increase of weight, and diminution of expectoration. In two of the cases recorded which were systematically treated for six or eight months the bacilli are said to have disappeared from the sputum.

Bouchard, whilst agreeing that inhalations and sprays of antiseptics benefit the patient in respect to weight and expectoration, makes the following weighty remarks: "I consider in a general way that external means are insufficient in so deeply seated a disease, the products of the breaking down of the lung may be modified, but what would be of great moment would be to be able to act in the thickness of the parenchyma itself in the heart of the tissues, where the tubercles are advancing in all their virulence, and not upon the détritus of softened tubercles, which are no longer harmful, since they are already outside the organism. All these sprays (biniodide of mercury, borate and benzoate of soda, creosote, &c.) perhaps cause antisepsis of the surface, but cease to have the least action at 1-10 mm. below. I add that the pulmonary ventilation being brought about everywhere by healthy parts, the medicating agent is carried by preference into regions where it is useless, and only to a very slight extent penetrates into those affected by disease."

This last objection must appeal to all, for the diminished entry of air into the tubercular parts of a lung is too well known a fact to require dwelling upon.

Still more heroic methods of treatment have been suggested for dealing with this fell disease. Drugs have been introduced by injection through the tracheal wall, so that they might trickle down into the finest ramifications of the bronchi. Direct injection into lung tissue has been tried, and yet success has not been obtained.

With regard to phthisis, we may then say, despite all efforts to combat the disease, but little in the way of cure can be obtained by the use of antiseptics.

In infective and zymotic diseases but little success has rewarded the labours of investigators, although so long ago as 1857 a movement in this direction was started by Dr. Polli. He attempted by means of sulphites to oppose the catalytic changes which were then supposed to take place in zymosis. His own results are said to have been extremely successful, but it is a question whether this was not due to the general treatment of the malady. We all know the extremely excellent results that all physicians get with their own line of treatment, which, however, so often fails in the hands of others. Common sense and careful and painstaking watching, and treatment of the symptoms as they arise on general principles, seems at present the only line to pursue in such cases.

Enterica is a malady where we have distinct localised lesions. A means of keeping sweet the sloughing intestinal ulcers "is a consummation devoutly to be wished," for the danger of such foci is apparent. Dr. Fergus, of Glasgow, has reported a case of a child of less than two years of age in whom great improvement resulted from the administration of half-grain doses of phenol. I myself am acquainted with a striking case, in which half-grain doses of phenol, combined with opium, appeared to have a most salutary effect in lowering the temperature, and generally improving the patient's condition. After the drug had been administered for a few days a dark discoloration of the urine suggested discontinuance. Upon omitting the antiseptic the temperature immediately rose, and again dropped upon re-exhibition of the drug. A most successful recovery took place.

Lemaire, Murchison, Wallace, Buchanan and Fuller do not consider that phenol has any effect upon the course of the fever, although sometimes it appears to relieve tympanitis. Bouchard speaks highly of internal antiseptics in all ulcerative conditions of intestines. With regard to their use he says they ought to be insoluble, in a state of fine pulverisation, and should be given in small and often repeated doses. He recommends naphthol, salicylate of bismuth and charcoal. From the numerous successful cases recorded of the antiseptic treatment of enterica we may certainly gather some hope. The results of such treatment are, to say the least, suggestive, but we cannot yet say that we have discovered the drug to be used. Nor do we find, upon comparing cases treated by other methods, that the antiseptic treatment at present holds forth such advantages as its supporters hoped for. Internal antisepsis is at present in its infancy, and time alone can develop its possibilities.

